# Migration, Crystallization and Dissolution Changes of Salt Solution with Color Rendering Property in Porous Quartz Materials

**DOI:** 10.3390/molecules25235708

**Published:** 2020-12-03

**Authors:** Jing Zhao, Hongjie Luo, Xiao Huang

**Affiliations:** 1 Ancient Ceramics Research Center, Shanghai Institute of Ceramics Chinese Academy of Sciences, Shanghai 201800, China; 2 Institute for the Conservation of Cultural Heritage, Shanghai University, Shanghai 200444, China; xhuang@shu.edu.cn

**Keywords:** color rendering tracing, copper sulfate, porous cultural relics, crystalline brine accumulation zone, salt crystalline zone

## Abstract

In order to visually display the migration and crystallization process of salt solution in porous cultural relics, copper sulfate solution with color rendering property was selected to record the migration, crystallization and resolution of salt solution in simulated SiO_2_ samples under different environmental conditions in real time through high-resolution recording system, scanning electron microscope system, salt phase X-ray diffraction system, and so on. The results showed the migration of salt solution in porous samples was related to the structural characteristics of the porous samples, the migration rate of salt solution, the evaporation rate and the change frequency of crystallization–resolution, etc., in which the large pore size of the sample, the higher the concentration and the faster migration and evaporation rate of salt solution, the greater the change rate of the brine accumulation zone or salt crystallization zone in the different porous samples. During the humidification–drying cycles of rainfall, the higher the cycle frequency of humidification–drying was, the higher the drying temperature was, the more frequent the crystallization-analysis change of salt in the salt-bearing sample was, and the more extensive the distribution of salt crystal zone was. This is the first time to visualize the salt belt by simulating the changing process of a salt solution with a color rendering property in porous samples. This has scientific theoretical guidance for the study of the migration–crystallization changes of soluble salts contained in porous silicate cultural relics. The visibility analysis results of multilayer salt crystal belts can also provide the preliminary basis for further effective desalination of salt bearing cultural relics.

## 1. Introduction

Due to natural erosion, environmental changes and other reasons, the preservation of silicate cultural heritage is not optimistic. Many diseases that threaten the safety of cultural relics have appeared, among which salt damage is the most common and serious disease [1]. For non- movable silicate relics (e.g., murals, grottoes, earthen sites, etc.), soluble salts migrate continuously under the action of capillary force formed by water in the voids of silicate materials, or due to concentration differences; when the concentration of the salt solution in a certain area reaches saturation, the salt crystallizes and expands, which leads to the fracture of the pore wall of the material [2,3,4,5,6,7,8,9,10,11].

Since the excavation of the Yungang Grottoes, a World Cultural Heritage Site, about 50,000 stone cultural relics have been damaged by various stresses in nature, and the most common is the destruction of the rock mass (including stone carvings) caused by the crystallization pressure of salt in the pores and voids of the rock [12,13]. Meanwhile, in the Mogao Grottoes, another World Cultural Heritage Site, within just a few decades, some murals have become blurred [14]. It has been confirmed that the repeated dissolution and expansion of soluble salt–crystallization shrinkage and the outward transport of soluble salts are the main reasons for pigment peeling off, loosening, falling or gradual dispersion [15,16,17,18].

At present, complex and precise instruments such as low field nuclear magnetic resonance and micro-CT are often used to detect and analyze the salt solution in porous cultural relics. However, there are some problems in the detection process; for example, hydrogen protons without salt ions were tested using low field nuclear magnetic resonance technology; considering the rate difference between salt ion and water ion in a salt solution, the tested result cannot reflect truly the process of salt ion migration. At the same time, the strict requirements of instrument detection for samples such as small size, uniform particle composition and non-magnetic, is difficult considering the complex composition and large volume of silicate grottoes, earth sites and so on. In all, it is difficult to observe the salt migration and concentration distribution of colorless salt solutions in the porous channels of samples under different environmental conditions.

In this study, we consider the soluble salt copper sulfate crystal with a color rendering property, as the anhydrous copper sulfate crystal is white, the copper sulfate solution and the copper sulfate pentahydrate crystal are blue with different hardness and morphology. Thus, through the comparison of color and microstructure, the migration, crystallization and dissolution processes of a salt solution in porous materials can be explicitly monitored.

## 2. Materials, Methods, and Experiments

### 2.1. Materials

Anhydrous cupric sulfate crystal (AR, purity: 99.0%, Shanghai Aladdin Biochemical Technology Co. Ltd. Shanghai, China), transparent PMMA box (cylinder with a diameter of 80 mm and cube with a thickness of 2 mm), porous SiO_2_ particles with the diameter of 0.105–0.71 mm, 1–2 mm, 2–3 mm, respectively (AR, Shanghai Aladdin Biochemical Technology Co. Ltd. Shanghai, China).

### 2.2. Methods

**Figure 1:**
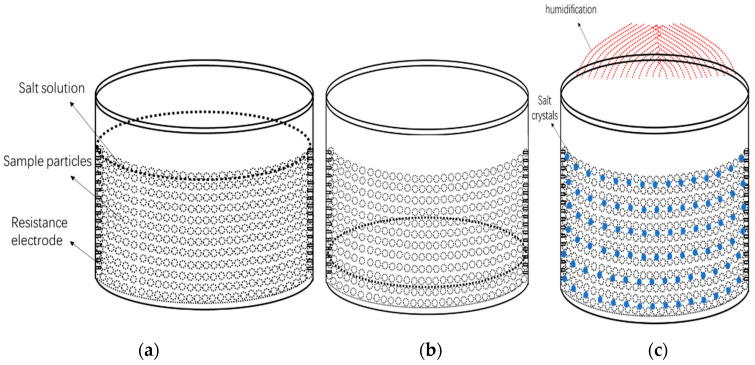
Schematic diagram of three types of simulation. (**a**) The salt solution in sample surface; (**b**) the salt solution cannot rise to the surface of the sample by capillary action; (**c**) simulated rainfall-drying process.

After taking the relationship between the salt solution and relic matrix in the natural environment into consideration, we simulated the migration and crystallization and other changing processes of salt solution in porous samples when the salt solution can rise to the sample surface, and the salt solution cannot rise to the sample surface. At the same time, in order to simulate the changes of environmental temperature and humidity of cultural relic samples with salt, especially the migration of salt solution during rainfall-drying, the overall design of the experiment included three types of simulation, as shown in Figure 1.

Figure 1a shows the state when the content of the salt solution was high and was on the surface of the sample; Figure 1b shows the state when the solution could not reach the surface of the sample through capillary action, and the content of the salt solution was relatively small, and the liquid was at the bottom of the sample; Figure 1c showed the changing state of the porous sample within the copper sulfate crystal after humidification–drying cycles.

As the particle size of porous samples affects its hydrodynamic characteristics to a great extent [18], the same SiO_2_ particles with different particle sizes were selected to simulate the changes of salt solution in the above three types of scenarios.

We prepared a 5.8% copper sulfate solution and weighed quartz materials with particle sizes of 0.105–0.71 mm (numbered B), 1–2 mm (numbered C) and 2–3 mm (numbered D), respectively. It was calculated that the content of soluble salt in different types of samples was 1.5%.

According to the size of quartz materials from small to large, the simulation samples in the first type were numbered as B1, C1 and D1, respectively; the simulation samples in the second type were numbered as B2, C2 and D2, and the simulation samples in the third type were numbered as B3, C3 and D3, respectively.

The simulated samples were placed in a cylindrical transparent glass box with a diameter of 80 mm, respectively, the ambient temperature was controlled, and the salt solution migration and crystallization changes of the simulated samples in the first type and the second type were recorded online in real time by video recorder (Sony FDR-AX45).

In the third type, the humidification–drying cycle of salt-containing simulated samples, humidification of 1% (m_water_/m_salt_ × 100%) was carried out on the surface of simulated samples with different particle size and the salt content accounting for 1.5% of the total weight of the samples, and the migration of the solution after humidification was observed. After this, the samples were dried in an oven at 40 °C or 80 °C for 8 h, respectively, and the migration process of salt solution in salt samples was observed.

### 2.3. Experiments

#### 2.3.1. Solution Surface Tension

On a KRUSS K100 mechanical surface tensiometer (Kruss GmbH, Hamburg, Germany), the surface tension of the salt solution was measured by the micro dispenser method; the standard deviation was 0.02 mN/m.

#### 2.3.2. Resistance Testing

A Keysight 344465A resistivity tester (Keysight Technologies, Santa Rosa, CA, USA) was used to test the resistance values of samples from different regions. The dissolution ratio was 22 bits, and DVC basic precision was 30 ppm.

#### 2.3.3. Morphology Testing

Compared with a conventional optical microscope, Japanese Keyeens’s ultra-depth-of-field microscope (with 54 million pixels) was used, and more than 20 times the depth of field observation was achieved. Morphology changes of salt in different samples were measured.

#### 2.3.4. Crystalline Phase Analysis

The crystal structure of the precipitated crystals was tested by Rigaku D/max 2550 V X-ray diffractometer (XRD). The working voltage and current were 40 kV and 100 Ma, respectively, and the measurement range of 2 θ was 5–80°.

#### 2.3.5. Permeability Testing

According to German industrial standard DIN52615, we controlled the steam flow naturally from high humidity to low humidity through the simulated SiO_2_ sample, then calculated the anti-steam vapor diffusion coefficient according to the formula:

μ = (P × δ_L_)/[M/(t × S × d)] (1)

in which t stands for time (h); M is the steam diffusing capacity (kg); S is the area (m^2^); P is the steam pressure under the test temperature (Pa); d is the sample thickness (mm); δ_L_ is the steam constant in the air = 7.02 × 10^−7^ (kg/m.h.Pa).

The greater the value of the anti-steam vapor diffusion coefficient μ is, the stronger the anti-steam diffusion ability is, and the worse the air permeability of the sample is.

The specific experimental methods were as follows: the polypropylene resin materials with permeability and hydrophobicity were sealed in a glassware carrying water, and then the simulated SiO_2_ samples were placed on the polypropylene resin materials, respectively, the samples were placed in the oven at 80 °C to test the quality changes in a different time, that was, the permeability of steam. Moreover, the anti-steam vapor diffusion coefficient μ value was calculated according to Formula (1).

## 3. Results and Analysis

### 3.1. Migration and Crystallization Changing Process of Salt Solution in Sample Surface

It was found that the cell volume of CuSO_4_ was 0.3633 nm^3^, and the cell density was 2.282 × 103 kg/m^3^. The surface tension of the CuSO_4_ solution with a concentration of 5.8% was tested to be about 73.72 mN/m at 22 °C. The crystal mode and the molecular cohesion of the solution surface tension were close to those of sodium sulfate crystal and solution.

The environment temperature and the relative humidity was controlled at 25 °C and 50%. Owning to the fact that the gravity and capillary force were the most important driving forces for the solution migrated in unsaturated porous media, the salt solution first moved down under the action of gravity in the porous SiO_2_ sample in Figure 1a, and the descent stopped at the action balanced; afterward, the solution migrated to the upper part of the sample as the capillary action of the solution.

**Figure 2:**
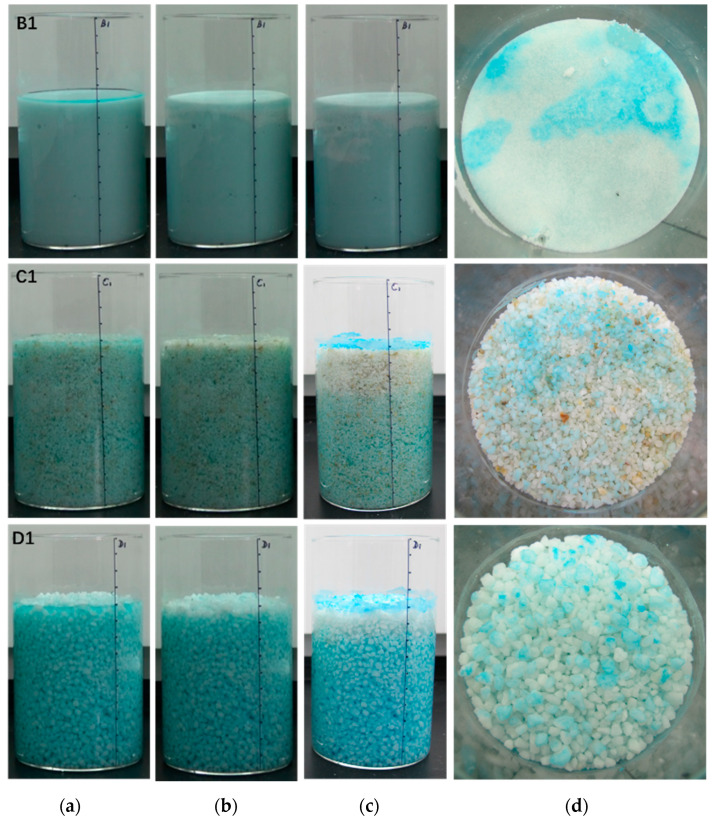
Process of change of salt solution in different samples. (**a**) Change of salt solution under the action of gravity and capillary force, placed for 0 h; (**b**) placed for 10 h; (**c**) placed for 40 h; (**d**) state of surface crystallization.

We compared the gravity water migration rate of B1, C1 and D1 samples with different particle sizes; the gravity water migration rate of C1 was the fastest (Figure 2). When the gravity drainage stopped, the salt solution moved continuously to the sample surface under the action of capillary force. With the continuous evaporation of the solution solvent water, the solution on the sample surface quickly reached saturation and began to crystallize. The changing process of salt solution in each sample is shown in Figure 2, and the crystallization state of the surface was also clearly visible.

### 3.2. Migration and Crystallization Changing Process of Salt Solution in the Interior of Sample

**Figure 3:**
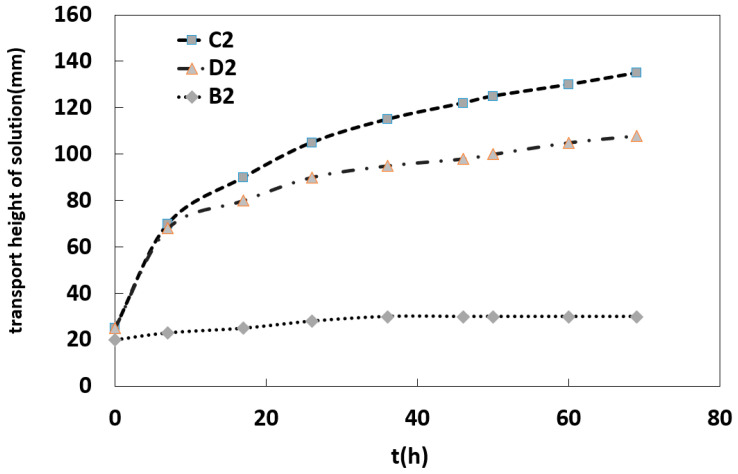
Capillary rising-rate curve of salt solution in samples.

The rising height of solution in the capillary could be calculated according to the Young–Laplace formula as h = $\frac{2\sigma \cos\theta }{r\rho g}$, where h is the rising height of solution in the capillary, σ is the surface tension coefficient of the solution, θ is the contact angle of the solution to the solid surface, r is capillary diameter, ρ is solution density, and g is the acceleration of gravity. It can be seen from the formula that the rising height of the solution in the sample capillary was limited; when the height of the simulated sample was greater than the elevated height of the salt solution capillary, it was observed that the rising height of the salt solution in the B2, C2 and D2 samples in Figure 3 was different. Among them, the rising height of salt solution in the B2 sample was the smallest, and the height remains stable after 30 mm. In contrast, the rising height of salt solution in C2 and D2 could reach 135 mm and 108 mm, respectively. Compared with the capillary rising-rate curve of salt solution in different samples, it can be seen that the capillary rising-rate of salt solution was the fastest for the SiO_2_ particles with the particle size distribution in 1–2 mm.

**Figure 4:**
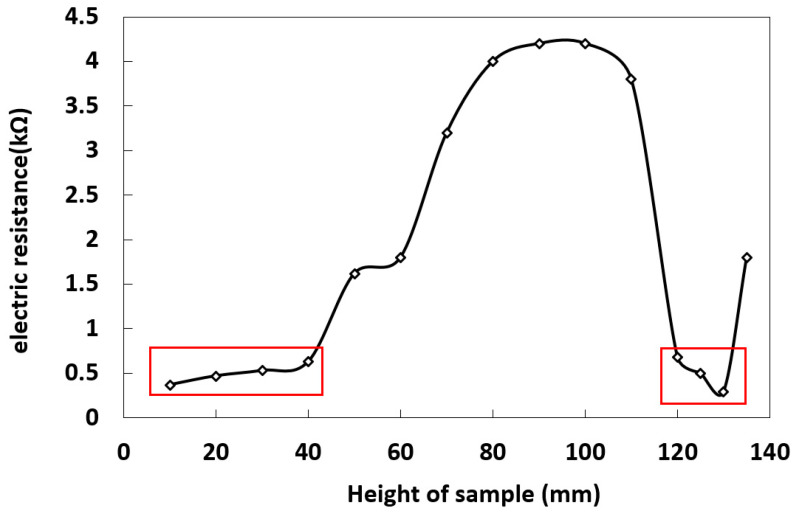
Solution resistance at different heights.

**Figure 5:**
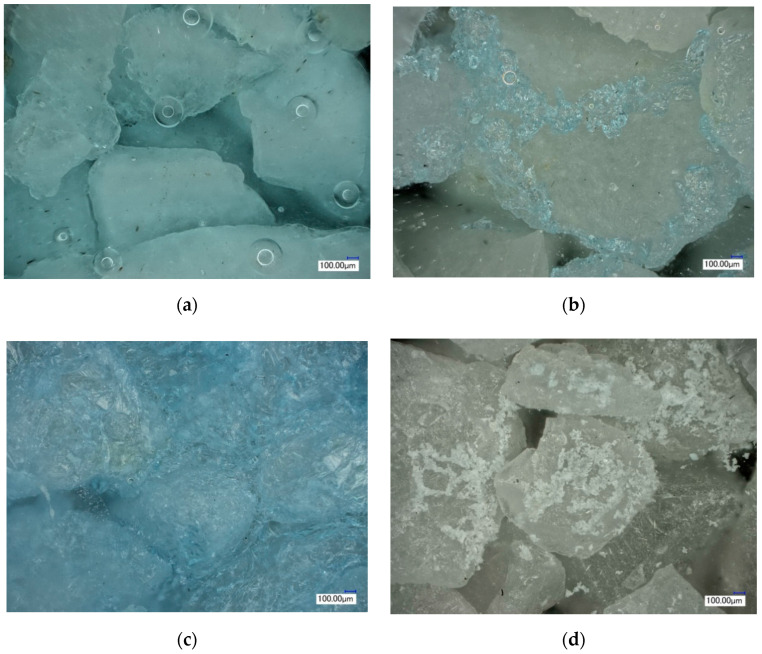
Observation of different forms of soluble salts in samples by ultra-depth-of-field microscope. (**a**) salt solution; (**b**) crystalline brine accumulation zone; (**c**) salt crystalline zone; (**d**) salt completely dried.

**Figure 6:**
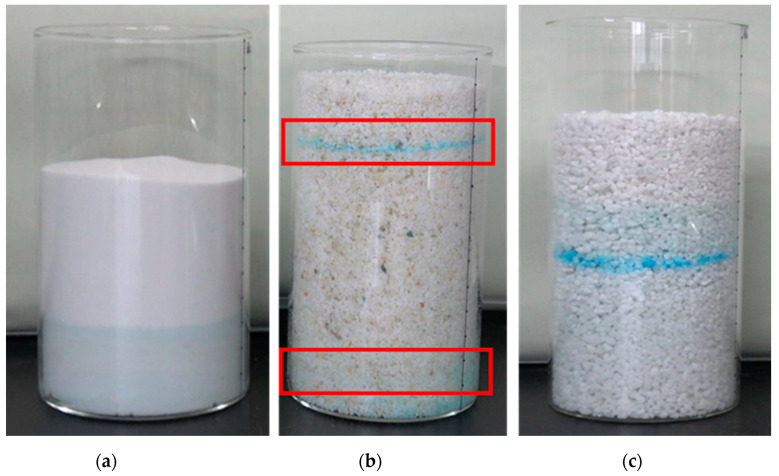
Migration crystallization state of salt solution inside samples. (**a**) B2; (**b**) C2; (**c**) D2.

In the process of salt migration with the continuous rise of capillary water, the electrical conductivity of the solution in different regions of the samples was tested by resistance electrode. The results showed that the concentration of salt ions in the solution decreased gradually with the increase of capillary water (Figure 4). When the capillary water rises to 120 mm, it was basically in a stable state; the blue crystalline brine water accumulation zone was formed about 24 h later (Figure 5b), and the resistance decreased rapidly. The specific changes in the sample are shown in the red boxes of Figure 6.

After the stable formation of the crystalline brine accumulation zone, due to differences between the external environment and internal samples, steam gradually evaporated in the crystalline brine accumulation zone; the stem formed in the evaporation zone entered the atmosphere through the dry area of the sample in the form of gaseous diffusion. Meanwhile, due to the loss of water, the salt solution gradually reached saturation, and the crystal precipitated, and a clear salt crystalline zone was formed in the interior, as shown in Figure 6c, and the specific microscopic morphology is shown in Figure 5c.

**Figure 7:**
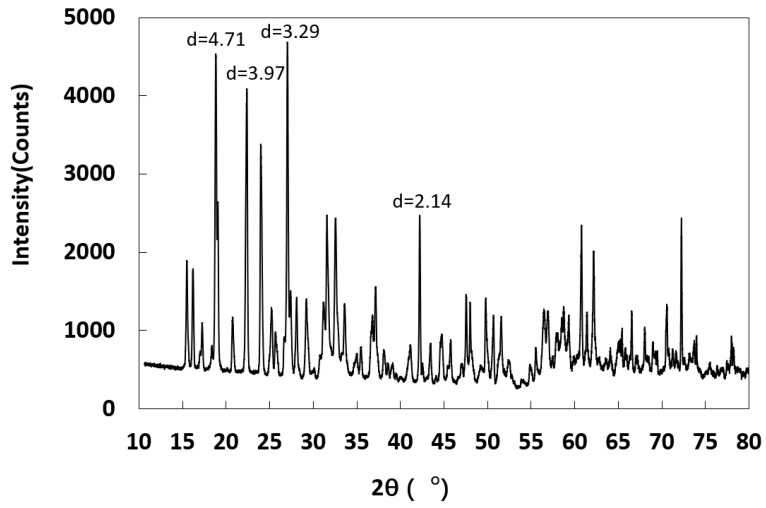
XRD curve of salt crystal zone in sample C2.

Compared with the salt crystalline zones of B2, C2, and D2 samples with the same external environmental conditions, the salt crystalline zone of the C2 sample was farthest from the bottom. The main reason for this is that the salt crystalline zone formed was related to the capillary water-carrying capacity of salt solution in the porous samples; the stronger the capillary water-carrying capacity, the higher the displacement of the salt crystalline zone from the bottom of the samples (Figure 6b). The crystal structure of the C2 salt zone is shown in Figure 7, which was mainly copper sulfate pentahydrate crystal adhered to the surface of quartz particles.

### 3.3. Effects of Simulated Rainfall-Drying Cycle on Salt-Containing Samples

**Figure 8:**
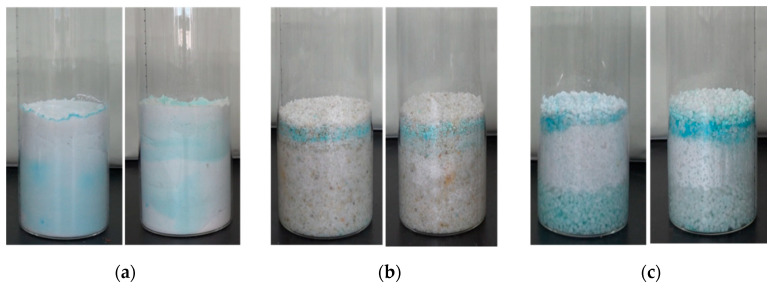
State of salt-containing samples after 1 and 3 humidification–drying cycles. (**a**) B3; (**b**) C3; (**c**) D3.

In the salt-containing sample, under the action of humidified water, which accounts for about 1% of the total mass of the sample, the internal salt was continuously dissolved and migrated to the bottom of the simulated sample under the action of gravity (Figure 8c).

When the salt solution rose continuously under the action of capillarity, and the ambient temperature increased, due to the difference between the surface steam pressure of the sample and the steam pressure in the ambient atmosphere, the steam on the surface of the sample diffused into the atmosphere under the action of the pressure gradient. This means that the evaporation of water on the surface of the sample was higher than the replenishment of water, which made the salt solution on the surface of the simulated sample saturated and crystals precipitated continuously.

When humidified again, the salt crystals on the simulated sample surface were partially dissolved, and the most significant molecular diffusion made the high concentration ions in the solution migrate to the low concentration, which is shown in the gradual dispersion of the salt accumulation zone in the figure.

As the convective transport of ions in the salt solution dissolved on the surface layer migrated to the interior of the sample, and the salt solution at the bottom of the sample migrated upward under the action of capillarity, a crystalline brine accumulation zone would be formed inside the sample when the water in the crystalline brine accumulation zone continued to evaporate. The crystalline zone was formed in the interior of the sample (Figure 8).

In the process of the formation of the crystalline zone, there were ion diffusion of the surface salt solution, capillary migration of the internal brine solution, the formation of the crystalline brine accumulation zone, and the formation of the salt crystalline zone after water evaporation; with the further partial dissolution of the formed crystalline zone, a multilayer or widened crystalline zone would be formed in the simulated sample.

The difference of samples B3, C3 and D3 under the same conditions mainly lies in the differences of ion diffusion, adsorption and migration rate of salt solution in different samples. The same mass of humidified water was injected into the sample, and the drop rate in the B3 sample was 1.21 mm/min. Due to the large water saturation of the B3 sample, the humidified water dispersed widely and did not completely reach the bottom of the sample. After a small capillary rise height (Figure 3) and the loss of water rate was 0.81 g/h, dry salt crystallization was dispersed.

In samples C3 and D3, the humidified water could quickly reach the bottom of the sample, and the liquid level of the solution in the sample rose continuously with the increase of the humidified water content, then the loss of water rate in samples C3 and D3 were 2.15 g/h and 2.58 g/h, respectively, the salt solution in the sample migrated and changes rapidly; the crystal zone formed by salt solution and the dissolution change of crystal zone was the most frequent. With the increase of the number of cycles, the width of the salt zone formed by salt crystallization–resolution changes obviously.

### 3.4. Influencing Factors for the Variation of Salt Zone in Simulated Samples

According to the above experimental results, it can be seen that the crystallization state of salt formed by the same concentration of the salt solution is different in different types of simulated SiO_2_ samples, mainly including the salt crystallization layer on the surface of the sample and the crystalline brine accumulation zone and salt crystalline zone inside the sample; according to the change of different environmental conditions, the salt crystalline zone in the sample could form multiple layers.

The factors affecting the salt zone change of the simulated sample were analyzed, including the structural characteristics of the simulated sample, the migration rate of salt solution, the evaporation rate and the change frequency of crystallization-analysis, etc. In order to avoid the uncertainty of the migration direction of the salt solution in the sample and to more intuitively show the influence of solution concentration and ambient temperature on the sample, the sample was further placed in a transparent glass box with a thickness of only 2 mm, and the changes of CuSO_4_ salt solution with the concentration of 5.8% and 12.3% at 40 °C and 80 °C were observed, respectively. Based on this comparison, the effects of SiO_2_ samples with different particle sizes, salt solution concentration, capillary migration rate and solution evaporation rate on the crystalline zone were to be found.

#### 3.4.1. Structural Characteristics of Simulated Samples

The specific surface areas of SiO_2_ samples with particle sizes of 0.105–0.71 mm, 1–2 mm and 2–3 mm were tested as 0.180 m^2^/g, 0.125 m^2^/g and 0.065 m^2^/g, and the water-holding capacity were 343 g/kg, 308 g/kg and 227 g/kg, respectively.

In Figure 3, the capillary rising rates of salt solutions with the same concentration in SiO_2_ samples with different particle sizes were compared and analyzed; it was found that the capillary rising-rate of SiO_2_ particles with a particle size of 1–2 mm was the fastest. As to whether the salt solution formed by the rising exists in the form of crystalline brine accumulation zone or salt crystallization, it is mainly related to the water evaporation of the crystalline brine accumulation zone.

**Figure 9:**
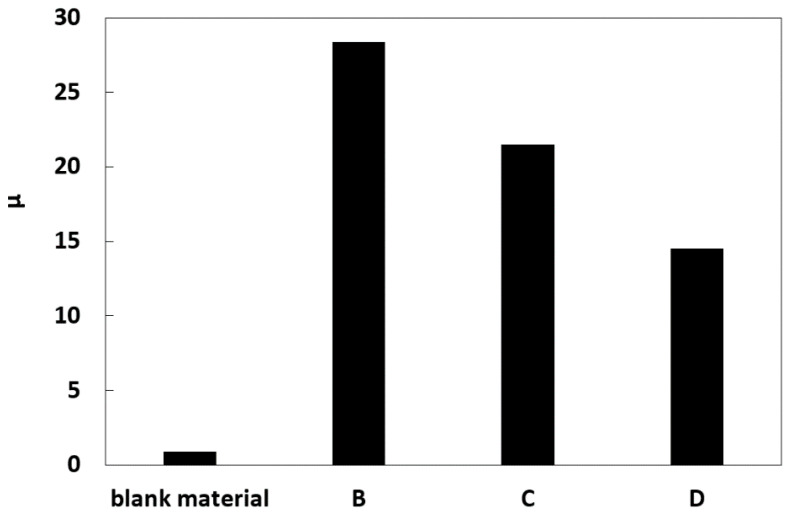
Anti-steam vapor diffusion coefficient in SiO_2_ samples with different particle sizes.

Among the simulated sample characteristics related to the water evaporation in the solution accumulation zone, air permeability was an important factor index; the vapor permeability of different samples was tested. In Figure 9, the anti-steam vapor diffusion coefficient of the carrier blank polypropylene resin material was the lowest, and the air permeability decreased by adding simulated SiO_2_ samples with different particle sizes, in which the air permeability of B2 material with the particle size of 0.105–0.71 mm was the worst. Compared to the results shown, the larger the particle size was, the better the steam permeability of the sample was. The salt crystalline zone was easy to be formed with the presentable air permeability in the crystalline brine accumulation zone of the sample, whereas the crystalline brine accumulation zone stably existed in the interior of the sample with the poor air permeability (Figure 6a).

#### 3.4.2. Migration Rate of Salt Solution

Considering that the capillary rising-height of salt solution in the simulated SiO_2_ sample with a particle size of 1–2 mm is relatively large, and the crystalline brine accumulation zone formed was also very obvious, the sample was further selected in the experiment. CuSO_4_ salt solution with a concentration of 5.8% and 12.3% were selected in the sample, with the serials numbers were I, II at 40 °C and III and V at 80 °C, respectively. The effects of different concentrations and ambient temperature on the migration of salt solution were compared.

**Figure 10:**
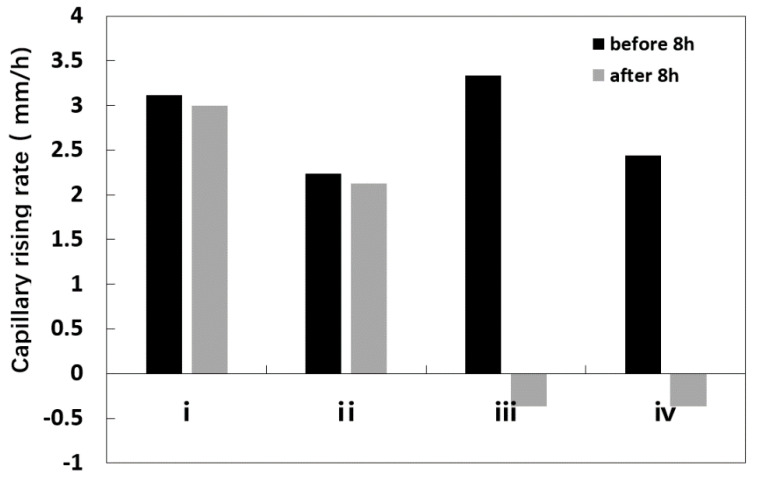
Capillary rising-rate of salt solution at different temperatures and concentrations.

The capillary rising and migration rate of salt solution in the sample was observed and calculated, as shown in Figure 10. The capillary rising-height and the capillary migration rate of the salt solution sample at 80 °C were larger in the first 8 h, and the capillary rising-height of the sample with a concentration of 5.8% was 89 mm, and the migration rate was 3.33 mm/h, the capillary rising-height of the salt solution sample with a concentration of 12.3% was 85 mm, and the capillary migration rate was 2.44 mm/h. The capillary rising-height of the salt solution sample with lower concentration was higher, and the migration rate of the solution was faster.

**Figure 11:**
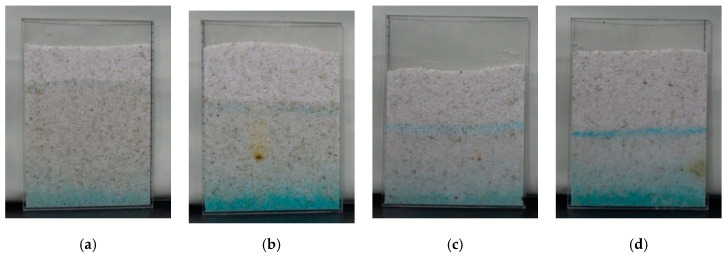
Surface state of the sample after being placed for 67 h. (**a**) i; (**b**) ii; (**c**) iii; (**d**) iv.

A visible salt solution crystalline zone was formed in the sample after 8 h (Figure 11). With the continuous evaporation of water in the salt solution crystalline zone, the zone gradually changed into the salt crystalline zone, and the capillary rising-rate decreased to a negative value.

Under the environmental conditions at 40 °C, the capillary migration height of the simulated sample with a salt solution concentration of 5.8% could reach 144 mm, and the migration rate is 3.00 mm/h, while the migration height of salt solution with a concentration of 12.3% in the sample was 120 mm, and the migration rate was 2.24 mm/h.

By comparing the same samples with different temperatures and different concentrations of salt solutions, it was found that the higher the ambient temperature was, the faster the capillary rise rate of the salt solution was, and the crystalline brine accumulation zone was soon formed, as shown in Figure 10d. With the evaporation of water in the crystalline brine accumulation zone, the crystalline brine accumulation zone was transformed into the salt crystalline zone. The lower the ambient temperature and solution concentration were, the higher the capillary rising-height of the salt solution were, as shown in Figure 10a, which was due to the fact that continuous migration of water ions carrying salt ions could rise to a higher height, thus forming an ion accumulation zone of the salt solution far from the bottom of the sample.

#### 3.4.3. The Evaporation Rate of a Salt Solution

**Figure 12:**
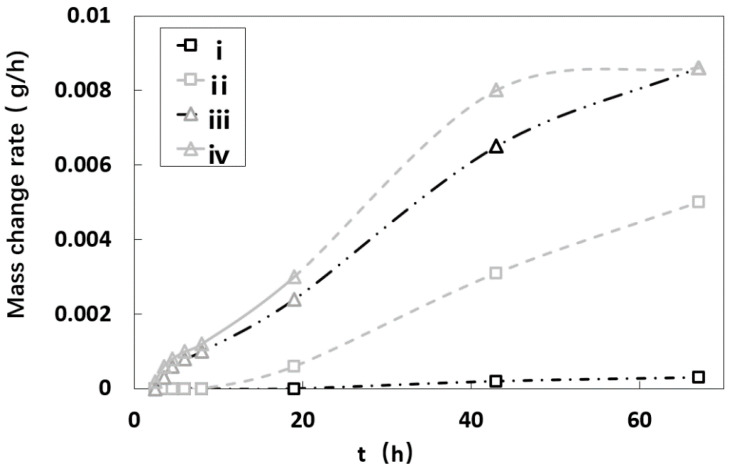
Evaporation rate curves of different samples.

By comparing the mass changes of SiO_2_ samples containing salt solutions of different concentrations under the conditions of 40 °C and 80 °C, it can be seen from Figure 12: there was a linear relationship between the change in mass and time of the samples with a salt solution at a concentration of 5.8% and 12.3% at 80 °C, respectively. The sample reached constant mass at 43 h and 67 h, respectively. The fitting curve equation for amount of evaporation of Sample iii and Sample iv was y = 0.0001x − 9E^−5^ and y = 0.0002x − 0.0002, and the correlation coefficients R² were 0.9888 and 0.9952, respectively. After comparing the slopes of the two equations, it was found that the higher the concentration, the greater the mass change rate at the same time.

Under the condition of 40 °C, the mass of the samples with the salt solution concentration of 5.8% and 12.3%, respectively, changed relatively slowly; the linear fitting curve equation of the change was y = 5E^−6^x − 3E^−5^ and y = 8E^−5^x − 0.0005, and the correlation coefficient R² was 0.9497 and 0.9852, respectively. Crystalline brine accumulation zone formed in the sample with a concentration of 5.8%.

The results showed that the higher the ambient temperature and the salt solution concentration was, and the higher the evaporation rate of the salt solution contained in the sample was.

#### 3.4.4. Cycle Frequency of Crystallization–Dissolution

When the salt crystalline zone was formed in the sample, the zone formed changes with the change of environmental rainfall-drying conditions. Due to the difference of capillary rising rate and evaporation rate, the salt-containing samples were dried at 40 °C and 80 °C, respectively, after humidification. The results showed that after the humidification of the sample and during the drying process of 40 °C, a small number of salt crystals precipitated on the surface of the sample due to the evaporation rate of salt solution, while during the drying process of 80 °C, a very obvious salt crystalline zone containing crystal water and dry crystal salt was formed on the surface and inside of the sample.

**Figure 13:**
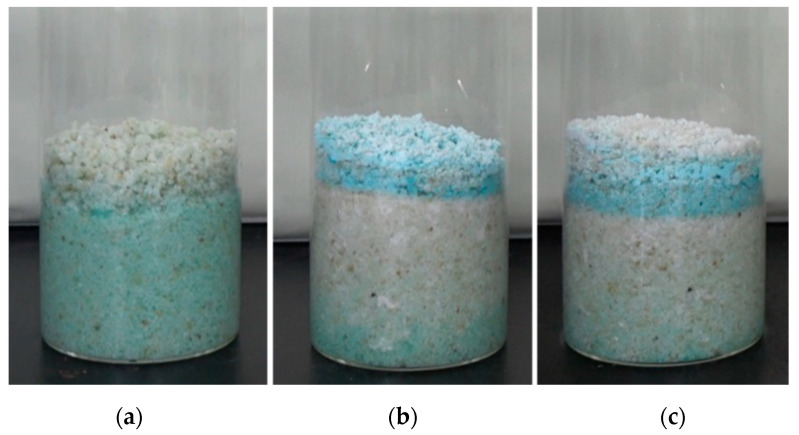
Sample changes at different drying–humidification cycle at 80 °C. (**a**) untreated; (**b**) after 1 cycle; (**c**) after 4 cycles.

During the process of dissolution, the dry crystalline salt on the surface of the sample dissolved quickly, and the salt solution migrated to the bottom of the sample under the action of gravity, resulting in an increase of solution content at the bottom of the sample as shown in the Figure 13. When dried again, the undissolved crystalline salt zone on the surface of the sample gradually lost water and dried, while the bottom solution migrated to the surface of the sample through capillarity, forming an obvious double-layer or even multilayer salt crystalline zone in the interior of the sample.

During the process of migration of brine solution, the actual migration of solute particles through pores was measured, and various physical and chemical phenomena occurring in pores macroscopically reflected hydrodynamic dispersion. The causes of hydrodynamic dispersion include the effects of solution flow, the complex microstructure of porous media, molecular diffusion, solution properties (density, viscosity) on migration. At the same time, its main function was the result of the simultaneous action of the material migration process, including convection, mechanical dispersion and molecular diffusion.

Among them, mechanical dispersion was a phenomenon of solute migration caused by the flow of solution and the existence of a pore system in which flow occurs, which made solute gradually spread to larger and larger flow area; whereas molecular diffusion is a phenomenon of material migration caused by the existence of concentration gradient in the solution, which made the substance of high concentration migrate to the place of low concentration in order to achieve the uniformity of concentration. In fact, hydrodynamic dispersion, including mechanical dispersion and molecular diffusion, plays an important role in the migration of salt solution and the formation of crystalline brine accumulation zone.

From the above results, it can be seen that the higher the temperature was, the faster the capillary rising-rate and water evaporation rate of the salt solution were, resulting in the formation of a salt crystalline zone within the sample, which will dissolve the salt ions in the further humidification process and lead to the further diffusion and migration of the salt solution under the action of hydrodynamic dispersion. The larger the cycle frequency of the crystallization-dissolution was, the more widely the salt crystals were distributed.

## 4. Conclusions

In this study, copper sulfate solution with color rendering property was used to record the migration–crystallization and dissolution process of salt solution in simulated samples under different environmental conditions online and in real time. The research results showed that:

(1) When the salt solution was on the surface of the sample, it would move towards the bottom of the sample under the action of gravity at first. When the gravity drainage stopped, capillary action becomes the main factor of the solution movement;
(2) When the salt solution could not rise to the surface of the sample through capillarity, it was easy to form a crystalline brine accumulation zone or salt crystalline zone in the simulated sample; the salt zone formed was mainly affected by the structural characteristics of the sample, the migration rate of the salt solution, the evaporation rate and the changing frequency of humidification–drying, in which the higher the ambient temperature, the faster the capillary rising-rate of the salt solution. A crystalline brine accumulation zone was quickly formed; with the evaporation of water in the crystalline brine accumulation zone, the crystalline brine accumulation zone was transformed into a salt crystalline zone. The smaller the particle size, the better the permeability, the lower the ambient temperature and the concentration of the salt solution, the higher the capillary height of the salt solution. This was due to the fact that the continuous migration of water ions and salt ions could rise to a higher height, thus forming a crystalline brine accumulation zone far from the bottom of the sample;
(3) During the humidification–drying cycle of simulated rainfall, the more frequent the humidification and drying cycle was, the higher the drying temperature was, the faster the capillary rise rate and water evaporation rate of the salt solution were, and the more frequent the diffusion and migration of salt ions within the sample was, resulting in the wider distribution of salt crystalline zone.

This is the first time to display visually the salt crystal zone, especially the multilayer salt crystal zone in the field of cultural relic conservation. In the past, it was mainly focused on the macroscopic observation of the surface salt or microscopic study of internal salts in the porous silicate samples, the formation of a macro salt zone and the visualization of the change of salt belt position have never been done before. It is of great significance to study the crystallization of salt in cultural relics, especially in large-scale immovable grottoes, murals and earthen sites. It also provides preliminary basic data for the study of the desalination of cultural relics.

## References

[1] Rörig-Dalgaard I. (2013). Development of a poultice for electrochemical desalination of porous building materials, desalination effect and pH changes. Mater. Struct..

[2] Dei L., Mauro M., Baglioni P. (1999). Growth of Crystal Phases in Porous Media. Langmuir.

[3] Noushine S.B., Salima R., Daniel B., Gerard W. (2008). Salt Crystallization during Evaporation, Impact of Interfacial Properties. Langmuir.

[4] Theoulakis P., Moropoulou A. (1999). Salt crystal growth as weathering mechanism of porous stone on historic masonry. J. Porous Mat..

[5] Espinosa-Marzal R.M., Scherer G.W. (2010). Advances in understanding damage by salt crystallization. Acc. Chem. Res..

[6] Espinosa-Marzal R.M., Scherer G.W. (2013). Impact of in-pore salt crystallization on transport properties. Environ. Earth Sci..

[7] Cardell C., Benavente D., Rodriguez-Gordillo J. (2008). Weathering of limestone building material by mixed sulfate solutions, Characterization of stone microstructure, reaction products and decay forms. Mater. Charact..

[8] Petković J., Huinink H.P., Pel L., Kopinga K., Van Hees R.P.J. (2010). Moisture and salt transport in three-layer plastersubstrate systems. Constr. Build. Mate..

[9] Rodriguez N.C., Linares F.L., Doehne E., Sebastian E. (2002). Effects of ferrocyanide ions on NaCl crystallization in porous stone. J. Cryst. Growth.

[10] Veran-Tissoires S. (2012). Salt crystallization at the surface of a heterogeneous porous medium. Europhys. Lett..

[11] Patricia V., Luque A., Francisco J. (2013). Surface changes on crystalline stones due to salt crystallization. Environ. Earth Sci..

[12] Ma Z.P., Huang J.Z., Zhang H. (2005). Study on chemical weathering of carbonate cements and related cultural relic diseases in Yungang Grotstone sandstones. Carsologica Sin..

[13] Yan S.J., Fang Y., Liu J.H., Tan S.E. (2013). Degradation test and model establishment of soluble salt on sandstone in Yungang Grottoes. Rock Soil Mech..

[14] Li Z.X. (2000). Present situation and tasks of the protection of Dunhuang Grottoes. Dunhuang Res..

[15] Disease Investigation Team of Dunhuang Research Institute and Disease Investigation Team of National Institute for Cultural Heritage, Tokyo, Japan (1992). A preliminary study on the causes of efflorescence diseases in No. 53 Cave of Mogao Caves. Dunhuang Res..

[16] Guo Q.L. (2009). Research on the Sources of Water and Salt in Dunhuang Mogao Grottoes Fresco Diseases. Ph.D. Thesis.

[17] Yu L.L. (2013). Research on the Occurrence and Development of Murals Diseases in Dunhuang Mogao Grottoes. Master’s Thesis.

[18] Jiang X. (2014). Study on the Capillary Transport Mechanism of Salt in Mural Wall Base. Master’s Thesis.

